# Prognostic value of neutrophil/lymphocyte ratio and mean platelet volume/platelet ratio for 1-year mortality in critically ill patients

**DOI:** 10.1038/s41598-020-78476-y

**Published:** 2020-12-09

**Authors:** Sung Yeon Ham, Hei Jin Yoon, Sang Beom Nam, Byung Hwan Yun, Darhae Eum, Cheung Soo Shin

**Affiliations:** 1 Department of Anesthesiology and Pain Medicine, Yonsei University College of Medicine, Seoul, Republic of Korea; 2 Anesthesia and Pain Research Institute, Yonsei University College of Medicine, Seoul, Republic of Korea; 3 Yongin Severance Hospital, Yonsei University College of Medicine, 23 Yongmunno, Yongin-si, Gyeonggi-do, 449-930 Korea

**Keywords:** Biomarkers, Medical research

## Abstract

Several studies have reported that the neutrophil to lymphocyte ratio (NLR) and mean platelet volume (MPV) are associated with poor prognosis. This study investigated whether NLR and/or the MPV/platelet ratio could function as predictive markers of mortality in critically ill patients. We retrospectively reviewed 1,154 patients admitted to the intensive care unit (ICU) between January 2017 and December 2017. Patients were divided into 2 groups according to 1-year mortality. We compared the NLR and MPV/platelet ratio on each day of ICU admission. Patients were classified into tertiles based on their NLR and MPV/platelet ratios, and the incidence of 1-year mortality was compared. Kaplan–Meier survival curves were plotted to evaluate their potential as prognostic factors for 1-year mortality. The NLR and MPV/platelet ratio were higher in the non-survivor group than in the survivor group. The incidence of 1-year mortality was the highest in the third tertile for both the NLR and MPV/platelet ratio. The MPV/platelet ratio was an independent predictor for 1-year mortality based on the Kaplan–Meier survival analysis. Our data showed that the MPV/platelet ratio is a predictive factor for 1-year mortality in critically ill patients.

## Introduction

The ability to predict a poor prognosis in critically ill patients is vitally important in determining a treatment strategy. Although several predictive tools have been suggested to predict mortality in critically ill patients, including the Acute Physiological and Chronic Health Evaluation (APACHE) II and the Sequential Organ Failure Assessment (SOFA), there are few predictive factors that are accurate, easy to use, and readily available in critically ill patients with various diseases^1,2^.

Most critically ill patients have cardiovascular instability and systemic inflammatory responses. For this reason, there has been extensive investigation into the prognostic abilities of various inflammatory biomarkers, including C-reactive protein (CRP) and procalcitonin, in critically ill patients^3,4^. However, there is an emerging need for a simple and readily available inflammatory marker such as the neutrophil to lymphocyte ratio (NLR) or mean platelet volume (MPV) to function as a prognostic indicator for patients in the intensive care unit (ICU). The NLR is a potent indicator of inflammation^5^, while the MPV is an indicator of platelet activation. In critically ill patients, inflammation causes vascular endothelial dysfunction in microcirculation as well as platelet activation and consumption. The NLR and MPV have been shown to be associated with poor prognosis in various cohorts, including those of patients with coronary artery disease, acute respiratory distress syndrome, cancer, and sepsis; those of elderly patients; and those of critically ill patients^6–10^.

Although the association between NLR and mortality in critically ill patients has previously been investigated, few studies have considered the prognostic values of both NLR and MPV.

This study aimed to investigate whether the NLR and MPV/platelet ratio could be used to predict 1-year mortality in critically ill patients.

## Results

**Figure 1:**
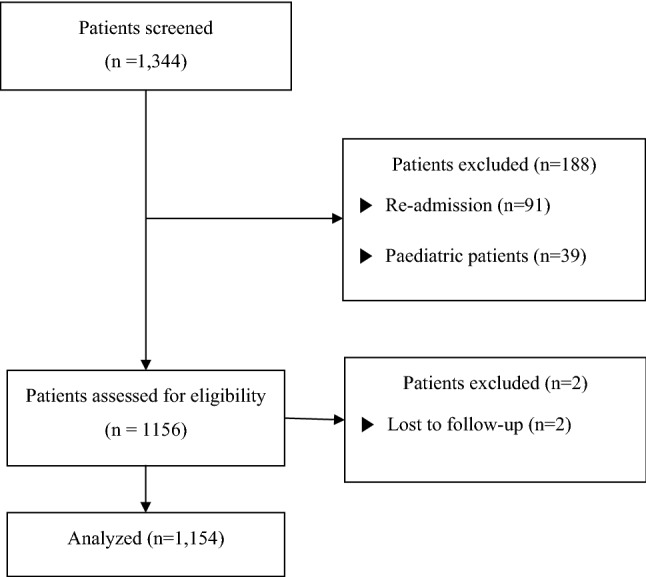
Flowchart of study enrolment.

A total of 1344 patients were screened, and 1154 patients were included in the final analysis (Fig. 1). Table 1 shows the baseline characteristics and clinical data of patients stratified according to 6-month mortality. Non-survivors were significantly older than survivors (68.49 ± 14.63 vs. 62.22 ± 15.88 years, *p* < 0.001) and the ICU readmission rate was significantly higher among the non-survivors than among the survivors (10.4% vs. 4.2%, *p* < 0.001). The survivor group contained a significantly higher proportion of postoperative patients than the non-survivor group (68.1% vs. 44.8%, *p* < 0.001). Severity of illness scores were significantly higher in the non-survivor group than in the survivor group. The APACHE II score was 14.91 ± 7.04 in the survivor group compared to 19.98 ± 9.03 in the non-survivor group (*p* < 0.001). The SOFA score was 3.89 ± 3.16 in the survivor group compared to 5.92 ± 3.80 in the non-survivor group (*p* < 0.001), while the Simplified Acute Physiology Score (SAPS III) was 27.21 ± 17.12 in the survivor group compared to 36.93 ± 21.43 in the non-survivor group (*p* < 0.001). The white blood cell (WBC) count was higher in the non-survivor group than in the survivor group (12,621.27 ± 6873.87 10^3^/µL vs. 11,676.43 ± 5932.20 10^3^/µL, *p* = 0.038). Conversely, haemoglobin (Hb) levels were significantly higher in the survivor group than in the non-survivor group (11.67 ± 6.00 g/dL vs. 10.46 ± 5.33 g/dL, *p* = 0.002). The neutrophil count was higher in the non-survivor group than in the survivor group (10,327.14 ± 5951.60 10^3^/µL vs. 9352.46 ± 5549.32 10^3^/µL, *p* = 0.015); in contrast, lymphocyte counts were higher in the survivor group than in the non-survivor group (1445.35 ± 1858.15 10^3^/µL vs. 1214.46 ± 1112.58 10^3^/µL, *p* = 0.011). The non-survivor group had a higher NLR than the survivor group (16.26 ± 25.42 vs. 10.18 ± 10.68, *p* < 0.001). The platelet count was not significantly different between the survivors (196.00 ± 89.81 10^3^/µL) and non-survivors (200.82 ± 146.45 10^3^/µL). The MPV and MPV/platelet ratio were both significantly higher in the non-survivor group than in the survivor group (MPV: 10.46 ± 1.13 vs. 10.16 ± 0.97, *p* < 0.001; MPV/platelet ratio: 8.97 ± 9.55 vs. 7.20 ± 11.78, *p* = 0.011). The length of ICU stay and duration of ventilator care were also longer in the non-survivor group than in the survivor group (ICU stay: 8.55 ± 13.28 days vs. 4.67 ± 18.38 days, *p* < 0.001; duration on ventilator: 6.02 ± 14.82 days vs. 1.96 ± 18.11 days, *p* < 0.001).

**Table 1:** Baseline characteristics and outcome variables stratified according to 1-year mortality.

	Survivor (n = 866)	Non-survivor (n = 288)	*p*-value
Sex (M/F)	517/349	179/109	0.461
Age (years)	62.22 ± 15.88	68.49 ± 14.63	< 0.001
Cancer (n)	109 (28.4%)	131 (34.0%)	0.104
Postoperative patients (n)	590 (68.1%)	129 (44.8%)	< 0.001
Emergency operation (n)	147/590 (24.9%)	33/129 (25.6%)	0.874
ICU readmission (n)	36 (4.2%)	30 (10.4%)	< 0.001
APACHE II score	14.91 ± 7.04	19.98 ± 9.03	< 0.001
SOFA score	3.89 ± 3.16	5.92 ± 3.80	< 0.001
SAPS III	27.21 ± 17.12	36.93 ± 21.43	< 0.001
Hb (g/dL)	11.67 ± 6.00	10.46 ± 5.33	0.002
WBC (10^3^/µL)	11,676.43 ± 5932.20	12,621.27 ± 6873.87	0.038
Neutrophil (10^3^/µL)	9352.46 ± 5549.32	10,327.14 ± 5951.60	0.015
Lymphocyte (10^3^/µL)	1445.35 ± 1858.15	1214.46 ± 1112.58	0.011
NLR	10.18 ± 10.68	16.26 ± 25.42	< 0.001
PLT (10^3^/µL)	196.00 ± 89.81	200.82 ± 146.45	0.598
MPV	10.16 ± 0.97	10.46 ± 1.13	< 0.001
MPV/platelet	7.20 ± 11.78	8.97 ± 9.55	0.011
Hospital stay (day)	25.04 ± 58.00	28.10 ± 27.85	0.388
ICU stay (day)	4.67 ± 18.38	8.55 ± 13.28	< 0.001
Ventilator day (day)	1.96 ± 18.11	6.02 ± 14.82	< 0.001

Values are presented as the mean ± standard deviation or number of patients (%).

ICU, intensive care unit; APACHE II, Acute Physiology and Chronic Health Evaluation; SOFA, Sequential Organ Failure Assessment; SAPS III, Simplified Acute Physiology Score; Hb, haemoglobin; WBC, white blood cell; NLR, neutrophil to lymphocyte ratio; PLT, platelet; MPV, mean platelet volume; HOD, hospital day.

Table 2 presents the categorisation of patients by baseline NLR tertiles, and Table 3 presents the categorisation of patients by the baseline MPV/platelet ratio. The highest severity of illness scores were observed in the third tertile (APACHE II score: 15.09 ± 7.47 vs. 15.68 ± 7.71 vs. 17.75 ± 8.25, *p* < 0.001; SOFA score: 3.97 ± 3.62 vs. 4.14 ± 3.13 vs. 5.08 ± 3.47, *p* < 0.001; SAPS III: 27.82 ± 17.47 vs. 28.36 ± 18.10 vs. 32.71 ± 20.25, *p* < 0.001). The length of ICU stay and duration of ventilator care were significantly longer in the third tertile (ICU stay: 4.48 ± 6.71 vs. 4.90 ± 7.81 vs. 7.53 ± 28.10, *p* = 0.030; ventilator duration: 1.83 ± 5.80 vs. 2.02 ± 6.50 vs. 5.06 ± 28.81, *p* = 0.015, respectively). The incidence of 6-month mortality was the highest in the third tertile for NLR (17.2% vs. 17.7% vs. 27.3%, *p* = 0.001). The incidences of in-hospital mortality and 1-year mortality were also the highest in the third tertile for NLR (in-hospital mortality: 11.2% vs. 11.9% vs. 21.0%, *p* < 0.001; 1-year mortality: 20.3% vs. 21.3% vs. 33.2%, *p* < 0.001).

**Table 2:** Baseline characteristics and outcome variables across NLR tertiles.

	1st tertile (n = 384)	2nd tertile (n = 385)	3rd tertile (n = 385)	*p*-value
Sex (M/F)	239/145	231/154	226/159	0.598
Age (years)	63.55 ± 16.00	63.31 ± 15.36	64.49 ± 16.07	0.549
Cancer (n)	109 (28.4%)	131 (34.0%)	135 (35.1%)	0.104
Postoperative patients (n)	238 (62.0%)	261 (67.8%)	220 (57.1%)	0.009
Emergency operation (n)	52 (21.8%)	62 (23.8%)	66 (30.0%)	0.111
ICU readmission (n)	21 (5.5%)	24 (6.2%)	21 (5.5%)	0.868
APACHE II score	15.09 ± 7.47	15.68 ± 7.71	17.75 ± 8.25	< 0.001
SOFA score	3.97 ± 3.62	4.14 ± 3.13	5.08 ± 3.47	< 0.001
SAPS III	27.82 ± 17.47	28.36 ± 18.10	32.71 ± 20.25	< 0.001
Hb (g/dL)	11.45 ± 5.45	11.80 ± 8.28	10.87 ± 2.14	0.084
WBC (10^3^/µL)	8841.46 ± 5067.20	11,822.81 ± 4770.51	15,070.82 ± 6877.90	< 0.001
Neutrophil (10^3^/µL)	5570.44 ± 3420.67	9720.99 ± 3957.66	13,485.24 ± 6136.57	< 0.001
Lymphocyte (10^3^/µL)	2178.07 ± 2679.12	1274.45 ± 561.96	712.71 ± 404.47	< 0.001
NLR	2.91 ± 1.33	7.87 ± 1.68	24.28 ± 22.48	< 0.001
PLT (10^3^/µL)	196.12 ± 96.40	204.32 ± 109.73	191.17 ± 113.20	0.225
MPV	10.17 ± 0.98	10.19 ± 1.00	10.34 ± 1.07	0.038
MPV/platelet ratio	7.73 ± 9.25	6.59 ± 4.33	8.62 ± 16.62	0.043
Hospital stay (day)	23.36 ± 34.52	25.28 ± 24.67	28.78 ± 79.73	0.344
ICU stay (day)	4.48 ± 6.71	4.90 ± 7.81	7.53 ± 28.10	0.030
Ventilator use (day)	1.83 ± 5.80	2.02 ± 6.50	5.06 ± 28.81	0.015
Mortality (n)	84 (21.9%)	93 (24.2%)	141 (36.6%)	< 0.001
In-hospital mortality (n)	43 (11.2%)	46 (11.9%)	81 (21.0%)	< 0.001
6-month mortality (n)	66 (17.2%)	68 (17.7%)	105 (27.3%)	0.001
1-year mortality (n)	78 (20.3%)	82 (21.3%)	128 (33.2%)	< 0.001

Values are presented as the mean ± standard deviation or number of patients (%).

ICU, intensive care unit; APACHE II, Acute Physiology and Chronic Health Evaluation; SOFA, Sequential Organ Failure Assessment; SAPS III, Simplified Acute Physiology Score; Hb, haemoglobin; WBC, white blood cell; NLR, neutrophil to lymphocyte ratio; PLT, platelet; MPV, mean platelet volume; HOD, hospital day.

**Table 3:** Baseline characteristics and outcome variables across MPV/platelet ratio tertiles.

	1st tertile (n = 384)	2nd tertile (n = 385)	3rd tertile (n = 385)	*p*-value
Sex (M/F)	219/165	239/146	238/147	0.273
Age (years)	62.28 ± 17.19	63.83 ± 15.49	65.24 ± 14.54	0.036
Cancer (n)	134 (34.9%)	117 (30.4%)	124 (32.2%)	0.406
Postoperative patients (n)	233 (60.7%)	260 (67.5%)	226 (58.7%)	0.030
Emergency operation (n)	49 (21.0%)	65 (25.0%)	66 (29.2%)	0.130
ICU readmission (n)	26 (6.8%)	16 (4.2%)	24 (6.2%)	0.256
APACHE II score	15.67 ± 7.49	14.88 ± 7.35	17.96 ± 8.48	< 0.001
SOFA score	3.78 ± 3.18	3.55 ± 2.88	5.85 ± 3.74	< 0.001
SAPS III	27.98 ± 18.39	27.17 ± 17.91	33.74 ± 9.31	< 0.001
Hb (g/dL)	11.48 ± 5.25	12.21 ± 8.36	10.43 ± 2.08	< 0.001
WBC (10^3^/µL)	13619.48 ± 6161.08	11,533.59 ± 5590.39	10,585.24 ± 6411.66	< 0.001
Neutrophil (10^3^/µL)	10,963.35 ± 5957.43	9337.61 ± 5234.55	8489.71 ± 5514.95	< 0.001
Lymphocyte (10^3^/µL)	1576.40 ± 1080.22	1415.14 ± 971.15	1172.12 ± 2557.89	0.004
NLR	10.83 ± 11.16	10.24 ± 10.60	14.01 ± 22.71	0.013
PLT (10^3^/µL)	306.83 ± 104.67	184.03 ± 25.68	101.03 ± 34.05	< 0.001
MPV	9.67 ± 0.72	10.15 ± 0.85	10.23 ± 1.02	< 0.001
MPV/platelet	3.37 ± 0.76	5.59 ± 0.69	13.96 ± 17.86	< 0.001
Hospital stay (day)	26.85 ± 35.38	20.31 ± 18.33	30.26 ± 80.75	0.001
ICU stay (day)	5.57 ± 9.24	4.11 ± 5.00	7.24 ± 28.03	0.004
Ventilator use (day)	2.81 ± 8.95	1.77 ± 5.47	4.33 ± 28.27	0.046
Mortality (n)	106 (27.6%)	74 (19.2%)	138 (35.8%)	< 0.001
In-hospital mortality (n)	51 (13.3%)	33 (8.6%)	86 (22.3%)	< 0.001
6-month mortality (n)	75 (19.5%)	51 (13.2%)	113 (29.4%)	< 0.001
1-year mortality (n)	98 (25.5%)	63 (16.4%)	127 (33.0%)	< 0.001

Values are presented as the mean ± standard deviation or number of patients (%).

ICU, intensive care unit; APACHE II, Acute Physiology and Chronic Health Evaluation; SOFA, Sequential Organ Failure Assessment; SAPS III, Simplified Acute Physiology Score; Hb, haemoglobin; WBC, white blood cell; NLR, neutrophil to lymphocyte ratio; PLT, platelet; MPV, mean platelet volume.

The APACHE II score, SOFA score, and SAPS III were the highest in the third tertile for MPV/platelet ratio (APACHE II score: 15.67 ± 7.49 vs. 14.88 ± 7.35 vs. 17.96 ± 8.48, *p* < 0.001; SOFA score: 3.78 ± 3.18 vs. 3.55 ± 2.88 vs. 5.85 ± 3.74, *p* < 0.001; SAPS III score: 27.98 ± 18.39 vs. 27.17 ± 17.91 vs. 33.74 ± 9.31, *p* < 0.001). The incidence rates of in-hospital, 6-month, and 1-year mortality were also the highest in the third tertile for MPV/platelet ratio (in-hospital mortality: 13.5% vs. 8.6% vs. 22.3%, *p* < 0.001; 6-month mortality: 19.5% vs. 13.2% vs. 29.4%, *p* < 0.001; 1-year mortality: 25.5% vs. 16.4% vs. 33.0%, *p* < 0.001).

**Figure 2:**
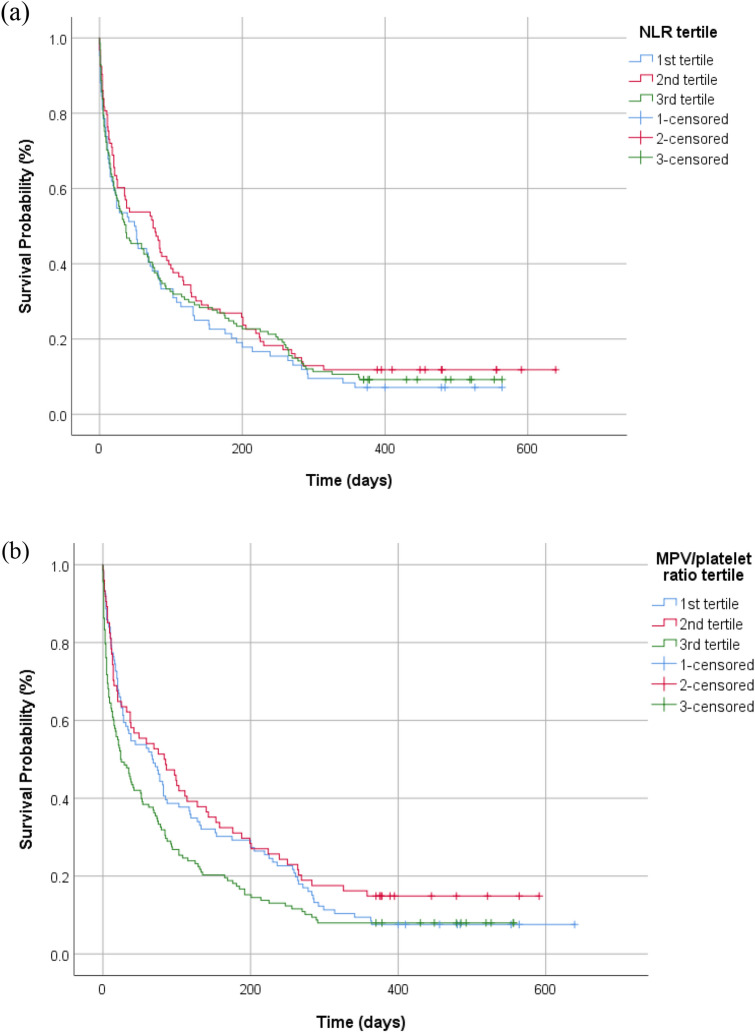
Kaplan–Meier survival curves for 1-year mortality according to the tertiles of neutrophil to lymphocyte ratio (NLR) (p = 0.498 based on the log-rank test) (**a**) and mean platelet volume (MPV)/platelet ratio (p = 0.015 based on the log-rank test) (**b**).

Based on the Kaplan–Meier curves for 1-year mortality, the log-rank test demonstrated that the MPV/platelet ratio was an independent risk factor for mortality in critically ill patients (*p* = 0.015). However, there were no significant differences in mortality between the tertiles of baseline NLR values based on the Kaplan–Meier curves (*p* = 0.498) (Fig. 2).

## Discussion

This retrospective study investigated the association between the NLR and MPV/platelet ratio and 1-year mortality in critically ill patients. We found that both the NLR and MPV/platelet ratio were significantly higher in the non-survivor group than in the survivor group. The 1-year mortality rate was significantly higher in patients in the third tertiles of both the NLR and MPV/platelet ratio than in patients in the lower tertiles. Moreover, the MPV/platelet ratio was independently associated with an increased risk of mortality at 1 year.

These findings are consistent with those of previous studies that identified the NLR as an independent predictor of mortality. NLR was demonstrated to be an independent predictor of mortality in patients with cardiovascular diseases such as acute coronary syndrome^6^, pulmonary embolism^11,12^, and aortic dissection^13,14^. It also has been shown to predict outcomes in cancer patients^8^.

The NLR increases in patients with neutrophilia and lymphopenia and is an indicator of systemic inflammation. NLR may reflect immune responses to surgical stress, systemic inflammation, or sepsis^5^. A previous study reported that neutrophilia and lymphopenia were observed in critically ill oncological patients following major surgery, sepsis, and septic shock and that there was a correlation between the severity of the clinical course and the grades of neutrophilia and lymphopenia^5^. This alteration in NLR is likely due to a stress-induced increase in serum cortisol levels. Furthermore, higher neutrophil counts induced by inflammation may contribute to the thrombotic state, explaining the utility of NLR as a predictor of poor prognosis^12^.

The role of NLR as a prognostic indicator in sepsis patients is controversial. A previous study on critically ill patients with sepsis found that there was no significant relationship between NLR and mortality. However, NLR was associated with mortality in unselected critically ill patients, including sepsis patients^15^. Additional studies have reported that NLR was associated with mortality in patients with sepsis^16–20^. Hwang et al. demonstrated that septic shock patients at risk of early death had a low NLR and that late death was associated with an increased NLR^21^. These disparate results in sepsis patients are presumably due to the presence of variables such as neutropenia, depending on the severity of sepsis. Consistent with this, an inverse pattern was observed in the NLR of patients at risk of early death compared to those at risk of late death^21^. Similarly, Hwang et al. reported comparable mortality rates in both the low and high NLR groups^16^. These finding are supported by previous studies demonstrating that neutropenia is a predictive factor for mortality in septic shock patients^22^. Therefore, our finding that NLR was associated with mortality in unselected critically ill patients, including sepsis patients, is consistent with previous results^15^. However, the use of NLR as a predictive factor in critically ill patients should be interpreted with caution given the possible involvement of neutropenia and neutrophilia in the case of sepsis, particularly since sepsis patients account for a high proportion of ICU patients.

Elevated MPV was described as an indicator of mortality in previous reports, consistent with our results. Previous studies were primarily concerned with the increase in MPV over time or after disease progression, rather than the initial value. MPV was significantly higher in the non-survivor groups than in the survivor groups after the third day of admission in a previous meta-analysis on noncardiac critically ill patients^23^. An increase in MPV during the first 72 h was an independent predictor for 28-day mortality in severe sepsis and/or septic shock patients^10^. The MPV/platelet ratio at admission and on day 1 was a prognostic marker for 28-day mortality in patients with severe sepsis, consistent with the findings of this study^9^.

MPV reflects the platelet size; therefore, an increase in MPV indicates an increase in platelet production and activation. Since platelet activation may contribute to the pathogenesis of the thrombotic complications of hypertension, platelet activation markers have been shown to increase with increasing severity of hypertensive disease^24^. Further, MPV was significantly higher in patients with coronary artery calcification than in patients without. As such, MPV has been revealed as an independent predictor of coronary artery calcification^25,26^. Elevation of MPV was also shown to be positively associated with arterial stiffness^27^.

MPV has been shown to be an indicator of inflammation in various diseases^28,29^. MPV was elevated in patients with malignant tumours compared to that in healthy subjects and decreased following treatment^30^. Elevated MPV was also associated with various cardiovascular diseases related to atherosclerosis, a disease that is characterised by endothelial dysfunction and vascular inflammation^27,31,32^. In critically ill patients, various conditions related to platelet activation that can lead to cardiovascular complications and inflammatory processes are important factors in determining prognosis. In this aspect, MPV can serve as a prognostic predictor for adverse outcomes in critically ill patients^33^.

Previous studies have shown that both MPV and MPV/platelet ratio have predictive values. The inverse relationship between the total platelet count and MPV has previously been described^10,34–36^. This inverse relationship was also observed in our study. When the patients were divided into 3 tertiles according to MPV, the platelet count decreased as the MPV value increased *(data not shown: 1st tertile, 238.00* ± *103.57; 2nd tertile, 190.75* ± *99.12; 3rd tertile, 159.10* ± *102.39; p* < *0.001)*. Azab et al. demonstrated that the MPV/platelet ratio was superior to MPV alone in predicting mortality in patients with non ST-elevation myocardial infarctions^37^. Therefore, our study examined the MPV/platelet ratio and revealed the value of the MPV/platelet ratio as a predictor of mortality in critically ill patients.

In this study, both the NLR and MPV/platelet ratio were significantly higher among survivors. Moreover, the incidence of 1-year mortality was significantly higher in patients in the third tertiles of NLR and MPV/platelet ratio than in those in the lower tertiles. However, based on the Kaplan–Meier survival curve, NLR was not an independent risk factor for mortality while MPV/platelet ratio did represent a significant risk factor for mortality in critically ill patients. These findings could indicate that both the NLR and MPV/platelet ratio are meaningful as predictors of mortality in ICU patients but that the MPV/platelet ratio is more valuable than NLR. This discrepancy could be due to the fact that NLR can be affected by changes in the neutrophil count and that MPV can reflect platelet activation. Given that both NLR and MPV reflect inflammation, it can function as a prognostic indicator. Further study will be needed to evaluate the differences between the NLR and MPV/platelet ratio as prognostic factors for mortality in critically ill patients.

The strength of this study was that we investigated the role of both NLR and MPV/platelet ratio as a prognostic predictor in critically ill patients, something that has not previously been given a great deal of consideration. Moreover, as revealed through the Kaplan–Meier survival analysis, we found that the MPV/platelet ratio, rather than NLR, was associated with patient survival in critically ill patients. The fact that previous studies mostly focused on the increase in MPV over time or after disease progression, rather than on the initial value, is another strength of this study. To our knowledge, few studies have investigated the predictive value of the initial MPV/platelet value. As revealed in this study, the ability to predict prognosis through a complete blood count, an easy, convenient, and readily available laboratory test, will help personalise treatments and improve the prognosis for each patient.

This study has several limitations. The first is the retrospective nature of the study. Furthermore, although we demonstrated the value of the NLR and MPV/platelet ratio as a predictor of mortality, we could not identify correlations with other inflammatory markers. Additional prospective studies investigating correlations with other inflammatory markers will be needed to obtain more conclusive results. Finally, although the study was conducted in ICU patients, the patient group was heterogeneous. We included all patients admitted to the ICU during the study period. Thus, the study included a variety of patients such as surgical patients, patients with sepsis, cancer patients, and pneumonia patients. Although it is meaningful to reveal the prognostic value of the NLR and MPV/platelet ratio in these various critically ill patients, further studies will be needed to elucidate the meaning of each in a more homogenous patient group.

In conclusion, this retrospective study revealed that the MPV/platelet ratio was independently associated with an increased risk of 1-year mortality in critically ill patients. Further, we determined that NLR and MPV/platelet ratio, an inexpensive and readily available marker at ICU admission, was a predictive factor for 1-year mortality in critically ill patients.

## Methods

This study was approved by the institutional review board of Yonsei University Health System, Seoul, Korea (IRB protocol No. 3–2018-0264). The requirement for informed consent was waived by the IRB. All methods and procedures were carried out in accordance with the relevent guidelines and regulations. The study was performed according to the tenests of the Declaration of Helsinki. We retrospectively reviewed patients admitted to the ICU between January 2017 and December 2017 (n = 1344). Patients who were re-admitted to the ICU in the same admission period (n = 91), paediatric patients (n = 39), and patients with insufficient data (n = 58) were excluded (Fig. 1). To assess the risk of mortality after ICU admission, patients were divided into 2 groups based on 1-year mortality (survivor: n = 866; non-survivor: n = 288). We compared the NLR and MPV/platelet ratio for each day of ICU admission. Patients were classified into tertiles according to the NLR (first tertile: NLR < 5.3 [n = 384]; second tertile: NLR ≥ 5.3 ≤ 11.3 [n = 385]; and third tertile: NLR > 11.3 [n = 385]) and MPV/platelet ratio (first tertile: MPV/platelet < 4.51 [n = 384]; second tertile: MPV/platelet ≥ 4.51 ≤ 7.07 [n = 385]; and third tertile: MPV/platelet > 7.07 [n = 385]).

We collected data on baseline patient characteristics including sex, age, history of readmission to the ICU, history of cancer, history of operation, severity of illness scores (including the APACHE II score, SOFA score, and SAPS III), laboratory data (including WBC count, Hb level, MPV, platelet count, neutrophil count, and lymphocyte count), and clinical outcomes (including hospital day, ICU day, and duration of ventilator use).

The NLR and MPV/platelet ratio measured on ICU admission were recorded. The baseline characteristics and clinical data, including those of NLR and MPV, were compared by dividing the patients into survivors and non-survivors according to 1-year mortality. Patients were categorised by tertiles of the baseline NLR and MPV/platelet ratio. The primary outcome was the incidence of 1-year mortality in patients admitted to the ICU. The secondary outcomes were in-hospital mortality and 6-month mortality.

Blood samples for laboratory counts were collected into tubes containing ethylenediaminetetraacetic acid (EDTA). To prevent EDTA-induced platelet swelling, platelet counts and MPV were analysed with ADVIA 2120 haematology analyser (Siemens, Forchheim, Germany) within 30 min of sample collection.

### Statistical analysis

All statistical analyses were performed using SPSS version 23 (IBM Corp, Armonk, NY). Normality was assessed using the Kolmogorov–Smirnov test. The independent t-test or Mann–Whitney test was used to compare continuous variables. To compare three groups, the one-way analysis of variance or Kruskal–Wallis test was used, as appropriate. Categorical variables were compared using the chi-squared or Fisher’s exact test. Demographic and clinical data are presented as mean ± standard deviation or frequency (%). Kaplan–Meier survival curves were created to assess the probability of survival across the baseline NLR and MPV/platelet ratio tertiles according to 1-year mortality. The tertiles were compared by the log-rank test. A p-value < 0.05 was considered statistically significant.

## Data availability

Materials, data and associated protocols are available.
